# Complete Closed Genome Sequences of a *Mannheimia haemolytica* Serotype A1 Leukotoxin Deletion Mutant and Its Wild-Type Parent Strain

**DOI:** 10.1128/genomeA.00417-15

**Published:** 2015-05-07

**Authors:** Michael P. Heaton, Gregory P. Harhay, Timothy P. L. Smith, James L. Bono, Carol G. Chitko-McKown

**Affiliations:** Meat Animal Research Center (USMARC), Clay Center, USDA, ARS, Clay Center, Nebraska, USA

## Abstract

*Mannheimia haemolytica* is a bacterial pathogen that secretes leukotoxin (LktA) which binds to leukocyte membranes via CD18, causing bacterial pneumonia in ruminants. We report the complete closed genome sequences of a leukotoxin mutant and its parent strain that are frequently used in respiratory disease studies.

## GENOME ANNOUNCEMENT

*Mannheimia haemolytica* is an opportunistic Gram-negative pathogen in ruminants and the major cause of severe, acute, hemorrhagic fibrinonecrotic pneumonia in cattle (1). The primary virulence factor of *M. haemolytica* is leukotoxin A (LktA), a member of the repeats-in-toxins family of proteins (2, 3). LktA is secreted directly from the bacterial cytosol to the extracellular space, where it binds to integrin beta-2 (CD18) on the membranes of neutrophils (4), causing lysis, necrotic cell death, acute inflammation, and lung injury characteristic of pneumonia (1).

The first isogenic leukotoxin deletion mutant (*lktA*) was reported in 1995 (5) and has been widely used to study leukotoxin function (6–30). The parent strain (89010807 N, *lktA^+^*) was isolated by the Oklahoma Animal Diseases Diagnostic Laboratory from a calf with severe fibrinous pleuropneumonia (6). Determining the complete genome sequences of these strains allows leukotoxin function to be interpreted in the context of their other genes, and those from other sequenced *M. haemolytica* strains (31–34). Here, we report the complete closed genome sequences of wild-type *M. haemolytica* 89010807 N *lktA^+^* and its deletion mutant *lktA*.

Frozen cultures were grown 16 h on brain heart infusion (BHI) agar plates at 37°C and 5% CO_2_, inoculated in 10-mL BHI broth, grown for 7 h without shaking, collected by centrifugation, and extracted with a blood and cell culture DNA kit (Qiagen, Valencia CA). Sequencing was performed on a Pacific Biosciences RSII instrument (Pacific Biosciences, Menlo Park, CA) with libraries prepared from manufacturer’s kits. Reads were error-corrected and assembled using a hierarchical genome-assembly process (RS_HGAP_Assembly.3 Protocol), which produced single large contigs that were validated and improved with Quiver (35). The error-corrected read coverage used for genome assembly was 17.4- and 17.9-fold with an average read length of 6,800 and 6,528 bp for *lktA*^+^ and *lktA* strains, respectively. Self-similarity dot plots of the consensus sequences revealed >6-kb overlap between the contig ends (>99% identity) indicating circular chromosomes. Redundant overlapping sequences were removed from the 3′ ends to generate circularized sequences. The origins of replication were assigned according to reference accession no. CP004752, and new linear chromosome models were generated. Circularization was enforced and junctions verified by remapping the reads with Quiver. The junctions were validated, additional sequence errors resolved, and assemblies generated with >99.99% accuracy.

The respective genome sizes of *M. haemolytica* strains USDA-ARS-USMARC 56470 and 56467 (89010807 N, *lktA^+^* and *lktA*) were 2,705,355 and 2,704,219, with CDS counts of 2606 and 2603; gene counts of 2749 and 2750; tRNA counts of 65; rRNA counts of 20; and a GC content of 41.0%. The 7.6-kb *lkt* operon was identical between strains, excluding the replacement of *lktA* with a beta-lactamase gene. The parent *lkt* operon was identical to *M. haemolytica* strains 183, 2286, D153, D174, and M42548 (CP004752, CP006619, CP005972, CP006574, and CP005383, respectively).

### Nucleotide sequence accession numbers.

Sequences of parent and mutant strains were deposited in GenBank under the accession numbers CP011098 and CP011099, respectively.
